# A Comparative Review of the Extrinsic and Intrinsic Factors Regulating Lactose Synthesis

**DOI:** 10.1007/s10911-021-09491-6

**Published:** 2021-06-14

**Authors:** Anna Sadovnikova, Sergio C. Garcia, Russell C. Hovey

**Affiliations:** 1 Graduate Group in Nutritional Biology, Physician Scientist Training Program, University of California, Davis, CA United States; 2 School of Life and Environmental Sciences, University of Sydney, Sydney, Australia; 3 Department of Animal Science, University of California, Davis, CA United States

**Keywords:** Glucocorticoid, Prolactin, Insulin, Mammary culture, Alpha-lactalbumin

## Abstract

Milk is critical for the survival of all mammalian offspring, where its production by a mammary gland is also positively associated with its lactose concentration. A clearer understanding of the factors that regulate lactose synthesis stands to direct strategies for improving neonatal health while also highlighting opportunities to manipulate and improve milk production and composition. In this review we draw a cross-species comparison of the extra- and intramammary factors that regulate lactose synthesis, with a special focus on humans, dairy animals, and rodents. We outline the various factors known to influence lactose synthesis including diet, hormones, and substrate supply, as well as the intracellular molecular and genetic mechanisms. We also discuss the strengths and limitations of various in vivo and in vitro systems for the study of lactose synthesis, which remains an important research gap.

**Supplementary Information:**

The online version contains supplementary material available at 10.1007/s10911-021-09491-6.

## Introduction

The synthesis of lactose by the mammary epithelium occurs through a unique and conserved pathway that also varies across species. In a previous companion review [1], we outlined the extramammary, intramammary, and intracellular processes that direct lactose synthesis and secretion. The principal mechanisms involved in these processes include factors, such as diet and hormones and those specific to the transcription and post-translational modification of α-lactalbumin (LALBA). Not surprisingly, various species have evolved different approaches to regulate lactose production, which underscores the importance of selecting the appropriate model(s) for translational studies. In this manuscript we use a comparative, cross-species approach to review the key regulators and control points that modulate lactose synthesis and, in the process, outline the strengths and limitations of different in vivo and ex vivo*/*in vitro methods that have been used to generate these data.

## Biphasic Regulation of Lactose Synthesis by Plasma Glucose Levels

Given our goal is to review the many different control points that regulate lactose synthesis, we start here by outlining the effect that glucose supply and availability can impart on the mammary epithelium. As described earlier [1], plasma glucose is the main precursor for lactose synthesis and plays a key role in determining milk volume. However, the effect of its availability on milk lactose yield or content is biphasic, as demonstrated through a range of studies in dairy cows using post-ruminal infusion of starch, glucose, or gluconeogenic precursors (i.e., casein) [2–5]. Of these, the most direct approach for studying the effect of plasma glucose on milk composition is close-arterial provision of different doses of glucose directly to the mammary gland [6].

At suboptimal plasma glucose levels, mammary blood flow becomes the primary driver of lactose production [5, 7–10]. For example, when undernourished lactating goats were infused with glucose, the yield and content of lactose in milk were highest when 50 or 60 g of glucose was infused per day. Specifically, when 60 g of glucose was infused, lactose content and yield increased to 48.1 mg/ml and 41 g from 46.4 mg/ml and 33.3 g, respectively, at baseline [6]. Mammary blood flow also increased in response to up to 60 g/d of exogenous glucose, then remained stable at levels of 80 or 100 g/d, while milk lactose content and yield decreased to baseline values (46.5 mg/ml and 35.1 g, respectively) at the 100 g/d dose [6]. Importantly, the level of glucose extracted by the mammary epithelium was constant across all doses. Likewise, the milk fat and protein content was not affected by the glucose dose [6]. During these states of adequate glucose availability there was also a parallel decrease in the level of glucose-6-phosphate in the mammary tissue and/or milk, as occurs in both rats [11, 12] and goats [6].

On the other hand, during states of excess glucose supply there is a shift toward the intracellular accumulation of glucose metabolites that can suppress lactose synthesis. When 80 g/d glucose was infused to the udder, goats transitioned from negative to positive energy balance, and a larger amount of glucose left the gland unused [6]. A parallel indication of this shuttling of glucose away from lactose synthesis is the accumulation of glucose-6-phosphate in the mammary epithelium or milk. In dairy cows receiving excess glucose via post-ruminal infusion, the concentration of glucose-6-phosphate in milk increased while that of glucose-1-phosphate decreased [5]. These changes may reflect the actions of insulin (INS) responding to increased plasma glucose levels, where INS is a strong negative regulator of phosphofructokinase, which would lead to an increase in glucose-6-phosphate levels [6, 13].

The role of plasma glucose concentration and supply in the regulation of lactose synthesis in lactating humans remains less clear. Certainly the negative effect of hypoglycemia on breast milk lactose and yield in the setting of a prolonged fast is well-established [14–16]. However, the question of how excess plasma glucose modulates lactose synthesis still requires investigation. Whereas Neville et al. concluded that the elevation of plasma glucose to 8 mmol/l for 4–6 h did not impact milk lactose content or the rate of lactose synthesis [17], this level of plasma glucose is within normal limits for postprandial glucose levels. In a subsequent smaller experiment with three breastfeeding humans producing less than 500 ml daily, elevation of plasma glucose levels to 8 mmol/l resulted in a numerical increase in the lactose concentration in milk, from 189 to 203 mmol/l [17]. In our view, these findings warrant further validation in a well-powered study to clarify how varying plasma glucose levels impact milk lactose yield or content in humans.

In summary, it appears there is a biphasic effect of plasma glucose levels on lactose synthesis across ruminant and non-ruminant species. It must be noted that the physiology, lactose precursor requirements, evolutionary adaptations, and milk composition of ruminants and non-ruminants differ, and specific conclusions regarding the mechanism in one species cannot be attributed to that of another. It is tempting to speculate that at suboptimal plasma glucose levels, mammary blood flow is the predominant player in the regulation of lactose synthesis, while at excess plasma glucose levels, the accumulation of intracellular intermediates in the MEC contributes to a downregulation of lactose synthesis. It remains to be determined how, at the genetic, biochemical, and cellular level, this occurs and whether insights into this mechanism can be harnessed for tailored interventions to improve outcomes for those with metabolic dysregulation (i.e., diabetes mellitus or ketosis).

## The Hormonal Regulation of LALBA and B4GALT1 Synthesis

As we discussed previously [1], the abundance of LALBA and β-1,4-Galactosyltransferase-1 (B4GALT1) is a key determinants for lactose synthesis, where their expression in the mammary epithelium is tightly regulated by critical hormones, including prolactin (PRL), glucocorticoid (GC), INS, triiodothyroinine (T3) and epidermal growth factor (EGF). Here we outline how these factors individually, or combined, can alter LALBA and B4GALT1 synthesis, with a prefaced overview of different in vitro systems that have been used to draw these conclusions.

### In Vitro Models for Studying LALBA and B4GALT1

Despite their widespread adoption and utility, as well as their essential role in defining biological mechanisms, various culture models face the significant limitation that they do not faithfully recapitulate the extent of lactose synthesis and secretion that occurs in vivo. In many ways, this longstanding conclusion is unsurprising given that MEC within the gland must coordinately associate with other epithelial cells, stroma, and the vasculature to achieve complete functional differentiation. While some aspects of differentiation such as the formation of dome-like structures do occur in primary cultures of MEC, the primary milk proteins they synthesize are caseins such as ß-casein (*CSN2*), but not LALBA, which emphasizes that these cultures are more representative of an early- to midpregnant state [18]. As pointed out by others, gene expression for CSN2 and the appearance of cytoplasmic lipid droplets do not reflect secretory activation (also known as lactogenesis II) [19–21]. One exception to these limitations is a system that used a > 2-week lag in culture, which conferred hormonal sensitivity to primary MEC that went on to synthesize and secrete LALBA into the medium (~ 1–10 ng/ml/day) [22].

Among the different ex vivo*/*in vitro systems available, different lines of evidence support that explanted mammary tissue best-approximates the in vivo state. When mammary glands from midpregnant or pseudopregnant mice were dissociated to acinar fragments or diced into explants, MEC maintained cell–cell associations, their cuboidal shape, and synthesized LALBA and lactose [23–25]. However, the use of mammary explants as a model faces certain limitations. Within hours of exposure to PRL, INS, and GC, explants from midpregnant mice had a transcriptomic signature similar to that recorded during secretory differentiation in vivo. By contrast, prolonged stimulation by a hormonal combination that would normally accompany secretory activation in vivo resulted in a transcriptomic signature that was vastly different from that described in fresh mammary tissue isolated from mice during early lactation [26]. A parallel challenge is sustaining the synthesis of milk components in fresh mammary tissue from lactating animals for more than a few hours in vitro*.* While the rate of lactose synthesis and secretion under these conditions can be sustained in the short-term, a decline in lactose synthesis thereafter likely reflects, at least in part, the high metabolic rate of MEC. For example, when mammary tissue was isolated from lactating guinea pigs, half of the lactose present in the tissue was released into the culture medium within 5 min. Following a washout phase, lactose secretion into the medium was then constant for up to 2.5 h, during which time the release of lactose was 2–3 mg/g tissue/h [27]. After 48 h however, *CSN2* and *LALBA* mRNA and protein levels in mammary tissue and MEC decreased precipitously, even in the presence of PRL, INS, and GC [23, 24, 28].

**Fig. 1:**
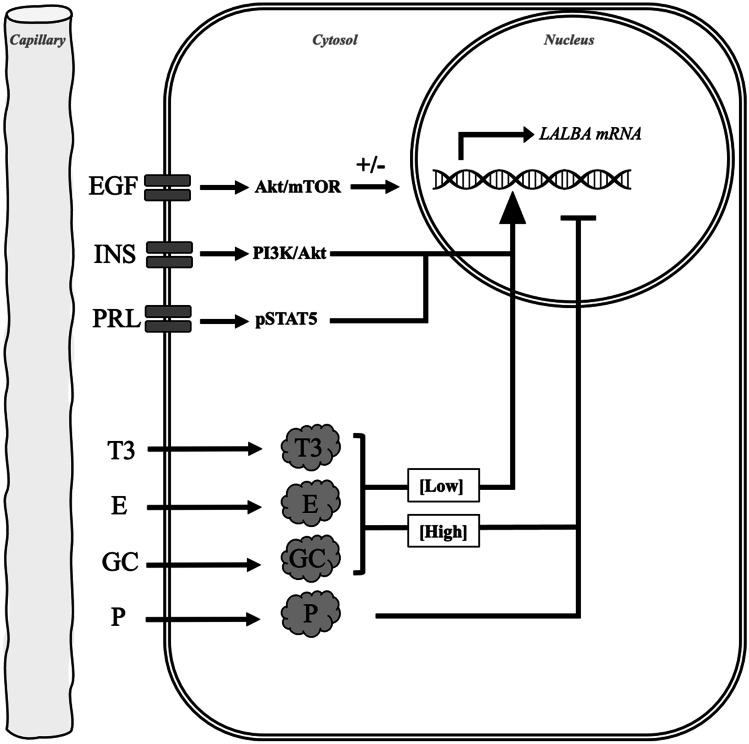
A schematic representation of the hormonal factors and mechanisms that regulate α-lactalbumin (LALBA) transcription. The positive regulators of LALBA transcription are prolactin (PRL) and insulin (INS). Progesterone (P) is a negative regulator of LALBA transcription. Thyroid hormone (T3), estrogen (E), glucocorticoids (GC), and epidermal growth factor (EGF) have variable effects on LALBA transcription that are species- or dose-dependent. Signaling occurs via intermediates including mammalian target of rapamycin (mTOR), phosphatidylinositol 3-kinase (PI3K), and phosphorylated signal transducer and activator of transcription 5 (pSTAT5)

Despite the limitations of these in vitro systems, they have certainly provided valuable insight into the hormonal regulation of LALBA and B4GALT1 gene and protein expression, as outlined in Fig. 1. In considering this summary, one should separately appreciate that the regulation of *LALBA* and *B4GALT1* expression by hormones in vivo may well differ from that in vitro given the presence of a potential myriad of physiologic influences including blood flow, nutrient supply, and varying hydrostatic and osmotic pressures. Moreover, cultured mammary tissue responds differently to hormones depending on the reproductive state of the donor [29, 30]. To assist the reader, we provide Supplementary File 1 that documents the culture conditions and outcome measures used in the literature that we describe hereafter.

### Prolactin

There is a clear requirement for PRL during the initiation of lactose and LALBA synthesis in most mammals, an effect that is most pronounced during the preparation for secretory activation. For example, in humans, plasma PRL levels peaked immediately after parturition, then fell to 50 to 100 ng/ml unless stimulated by suckling or pumping [44]. This transient elevation of plasma PRL levels was associated with a 3.5-fold increase in *LALBA* mRNA in the milk fat globule membrane after 6 h [45]. In a range of species including non-pregnant humans and non-human primates, the synthesis of lactose alongside milk secretion can also be induced by exogenous estrogen (E) and progesterone (P) combined with a PRL-secretagogue [31–33]. Lactose synthesis was also induced in pseudopregnant mares and rabbits treated with a PRL-secretagogue or recombinant PRL, respectively. By contrast, endogenous PRL was sufficient to induce lactose synthesis in pseudopregnant heifers and goats [34–37]. Exposure of pregnant gilts to the PRL-secretagogue domperidone in late pregnancy also tended to increase the abundance of *LALBA* mRNA in biopsied mammary tissue by day 2 postpartum [38].

The effect of PRL on lactose synthesis during established lactation is less pronounced. Domperidone administered to lactating dairy cows did not promote lactose synthesis [39, 40], while milk lactose content was increased in lactating dogs treated with the PRL-secretagogue, metoclopramide, during the first week of lactation [41]. Both domperidone and metoclopramide are prescribed to increase milk production in humans although the quality of evidence supporting their effectiveness is low [42]. Administering recombinant PRL to lactating humans with PRL deficiency or to those pumping for their premature infants increased milk lactose content from 53 to 63 mg/ml, and the concentration of neutral and acidic oligosaccharides doubled without affecting milk fat or protein content [43].

The positive effect of PRL on lactose synthesis occurs primarily at the level of *LALBA* and *B4GALT1* transcription, where PRL first binds the PRL receptor to activate the interconnected Janus kinase 2/Signal transducer and activator of transcription 5 (STAT5) and phosphatidylinositol 3-kinase/Akt signaling pathways (Fig. 1). Phosphorylated STAT5 then binds other transcription factors such as the GC receptor (GR) prior to recruitment to promoter and enhancer regions of milk protein genes [46–48]. The critical role of PRL-induced signaling in lactose synthesis is highlighted by the fact that lactating mice with a conditional deletion of STAT5 within their mammary glands had decreased expression of *LALBA*, but not *CSN2* [49].

It is worth highlighting that the stimulatory effect of PRL on LALBA synthesis occurs in concert with other lactogenic hormones, specifically GC, INS, and T3. The synthesis of CSN2 and lipid in mammary explants from midpregnant mice and rats required PRL [50, 51]. Whereas LALBA could also be synthesized by explants cultured in a medium supplemented with only INS and GC, its synthesis was delayed by 24 h in the absence of PRL [52]. Similarly, in multi-week cultures of MEC from virgin and pregnant rats, the combination of INS and GC also stimulated LALBA synthesis, albeit to levels that were 18-fold lower than those achieved by the combination of INS, GC, and PRL [22]. Likewise, the synthesis of LALBA in mammary explants from pregnant pigs was induced by PRL alone in a dose-dependent manner, while maximal LALBA synthesis required the combination of PRL, INS and GC [53].

The concentration of PRL required for maximal induction of LALBA synthesis in vitro also depends on the dose of GC, an effect that seems to be species-specific. Maximal LALBA synthesis by mammary explants from midpregnant rats occurred in the presence of a low concentration of GC (10 ng/ml) added to medium containing INS along with supraphysiologic levels of PRL (5 μg/ml). By contrast, when explants were cultured with a higher concentration of GC and PRL, as found in rats during late pregnancy (40 ng/ml and 1 ug/ml, respectively), the synthesis of LALBA was similar in the presence or absence of PRL [54]. The induction of LALBA by PRL in mammary tissue from midpregnant rabbits was enhanced by supplemental INS, but not GC [55].

Not surprisingly, an additional determinant of the extent to which LALBA synthesis responds to PRL is the reproductive state of the animal. Explants from postmenopausal humans required supraphysiologic concentrations of PRL (20 μg/ml) to initiate lactose synthesis, whereas a lower concentration (2 μg/ml) of PRL was required for the same response by explants from premenopausal individuals [29]. Likewise, induction of lactose synthase (LS) activity in mammary explants from virgin mice required either more time, or supraphysiologic concentrations of INS, PRL, and GC, to reach a short-lived peak in activity relative to the times and concentrations required for explants from pregnant or parous mice. When PRL, GC, and INS were added to mammary explants from parous mice, the ED_50_ of these hormones that was required to induce LS activity was much lower than the ED_50_ for CSN2, implying that parity conferred a lower threshold for hormone-induced activation of LS [56].

The in vitro sensitivity of B4GALT1 levels to PRL matches changes in the concentration of B4GALT1 and PRL around the onset of lactose synthesis in vivo, and consistently differs from that for LALBA. When PRL was added to mammary explants from midpregnant mice and rats, lactose synthesis first increased after 4 to 8 h [54, 57]. By contrast, LALBA activity in mammary explants from midpregnant mice increased after 18 h [57, 58]. The activity of B4GALT1 and LS in explants from midpregnant mice reached a maximum after 3 d in culture, concomitant with the secretion of lactose into the medium, whereas LALBA activity continued to rise until it peaked on day 6 of culture, by which time the secretion of lactose had decreased to its nadir and the activity of B4GALT1 and LS was low [57, 58]. In a similar way, PRL was only able to induce a constant, linear rate of LALBA production in mammary explants from midpregnant rabbits after a 1–2 day lag [55].

We should point out that this apparent asynchronous induction of B4GALT1 and LALBA by PRL in mammary explants from midpregnant mice should be interpreted with caution. Specifically, at a physiologic concentration of PRL (50 ng/ml), the upregulation of B4GALT1 and LALBA in response to PRL were comparable [59]. Furthermore, other experimental conditions, such as the GC concentration, could explain a twofold greater induction of B4GALT1 by PRL, given that a high GC dose (5 ug/ml) was later found to specifically inhibit LALBA synthesis [60].

In summary, while PRL clearly directs the upregulation and maintenance of lactose synthesis, there are undoubted species- and concentration-specific differences in how PRL regulates *LALBA* expression, as well as how it cooperates with other hormones such as INS and GC. Some of these mechanisms still lack resolution. Moreover, there are still gaps in our understanding of how downstream effectors of PRL signaling cascades, in concert with other hormone-regulated pathways, regulate the expression of *LALBA* and *B4GALT1* at the genomic level.

### Thyroid Hormone

The ability of thyroid hormones to regulate lactose and milk production has been the subject of inconsistent investigation over several decades. Oral or intranasal thyroid hormone releasing hormone (TRH) administered to breastfeeding individuals for 4 weeks postpartum increased PRL secretion, milk production, and in some cases milk lactose content without a change in milk protein or fat content [61–64]. The greatest positive impact of TRH on milk production and lactose content was among those with insufficient milk production who received TRH in the first week postpartum [61–64]. Conversely, lactation failure can be an early clinical manifestation of both hyper- and hypothyroidism [65].

The effect of hypo- or hyperthyroidism on lactose content in other species is less clear. Administering thyroxine (T4) to lactating cows increased daily lactose yield by 25% and milk lactose content from 52 to 54 mg/ml [66, 67]. Even though T4 is essential for the galactopoietic effects of PRL in mice [68], there is limited data to support whether exogenous T4 affects their milk lactose content. The induction of hypo- and hyperthyroidism during lactation variably affected lactose synthesis in rats [69, 70], where hypothyroidism lowered the milk lactose concentration on day 15 of lactation (L(15)), but was without effect on L(1) or L(21) [71].

Combined lines of evidence suggest that the positive effects of triiodothyronine (T3) or T4 on lactose synthesis are species-specific and occur through a direct effect on LALBA transcription. For example, adding T3 or T4 to cultured primary MEC from virgin or midpregnant rats did not stimulate LALBA synthesis [22], whereas others recorded a clear stimulatory effect of T3 on LALBA and lactose synthesis in explants of mammary tissue from mice [69, 70]. The level of *LALBA* mRNA and protein increased twofold in mammary tissue from midpregnant mice in response to T3, whereas levels of mRNA for *B4GALT1,* CSN2 content, total RNA, and total protein synthesis were unaffected [72, 73]. Whether T3 increased LALBA synthesis by exclusively stimulating transcription or extending the half-life of the *LALBA* mRNA transcript was not resolved [74].

One additional consideration is that T3 may modulate the actions of other hormones on MEC. Adding PRL to culture medium containing INS, GC, and T3 increased *LALBA* expression in murine explants by 40% above the level measured in cultures without T3 [72]. Whereas mammary tissue from virgin and midpregnant mice typically required supraphysiologic doses of INS, GC, and PRL over three days to induce LS activity, supplemental T3 or T4 reduced the necessary dose of INS, GC, and PRL to physiological levels, and increased lactose synthesis threefold [56, 72]. Over a range of concentrations, the L-forms of T3 and T4 were most stimulatory for LALBA synthesis, where the threshold for the induction of LALBA synthesis by L-T3 (10^–10^ M) was lower than for L-T4 (10^–8^ M) [72].

The regulation of lactose synthesis by thyroid hormones spans multiple levels and physiologic states and warrants continued investigation. In particular, the role of thyroid hormones in lactose synthesis is undoubtedly relevant for breastfeeding humans with thyroid disorders and clinical conditions involving metabolic dysregulation, such as obesity, as well as for high-producing dairy livestock that are prone to extreme negative energy balance. These questions also extend to the molecular level, where the action(s) of thyroid hormones on various milk protein genes, including LALBA, remain to be defined.

### Progesterone (P)

The role for P during the initiation of lactation is clear, where its circulating levels must decrease to initiate the onset of copious lactose synthesis during secretory activation. This critical role for P is highlighted in postpartum humans with retained placental fragments, where secretory activation was delayed until the P-secreting placental tissue was removed [75, 76]. The best demonstration of a mechanistic relationship between circulating P and the onset of secretory activation is the rapid induction of B4GALT1 and LALBA activity in mammary tissue homogenates isolated from rats following ovariectomy-induced depletion of P on day 19 of gestation [77]. This induction could be reversed when P was administered immediately after ovariectomy, whereas its inhibitory effect was less following administration 12 or 24 h later [77]. The effect of bilateral ovariectomy on total lactose content in mammary tissue was also evident in late-gestation rats 24 to 48 h after surgery, a response that was greater in rats ovariectomized later in gestation [78].

The mechanism by which P inhibits lactose synthesis primarily involves its repression of *LALBA* transcription. Notably, this repression is most pronounced in mammary tissue from preparturient animals and is species-specific. For example, whereas P inhibited LALBA synthesis in mammary explants from virgin and early- to mid-pregnant mice, the same dose only inhibited LALBA production by 50% in mammary tissue from late-pregnant rats. In lactating rats the effect of P on LALBA synthesis was less, where a 1000-fold higher concentration of P was required to decrease LALBA content in mammary tissue from lactating versus non-lactating rats [30]. By contrast to these findings for rats, the P-induced suppression of *LALBA* in explants from lactating, non-pregnant cows was more sensitive than was *CSN*2 or genes required for fat synthesis. For example, only 15 μM of P was required to inhibit *LALBA* transcription, whereas doses > 30 μM were required to inhibit *CSN2* transcription [79].

### Estrogens (E)

There are several indications that E can inhibit lactose synthesis during established lactation. Birth control pills delivering E + P decreased the content of LALBA in breast milk and overall milk production, although the volumetric decrease was still within the normal range of output [80]. Birth control pills containing E are also used to treat hyperlactation in humans, although the mechanism is undefined [81]. In lactating cows, a single dose of synthetic E accelerated mammary involution coincident with a reduced concentration of LALBA and lactose in milk following final milk removal [82, 83]. In a similar way, a high concentration (30 μM) of 17-β-estradiol inhibited LALBA secretion by mammary explants from lactating cows by 35–45% [79]. When high doses of E were administered to goats during midlactation, they demonstrated a varied response in milk composition, with most having a progressive decline in milk yield. Among those goats, two animals had complete suppression of milk and lactose production within four days [84].

Beyond these responses, there is also evidence for a biphasic effect of E concentrations on LALBA and lactose synthesis. For example, a low dose (50 μg) of synthetic E increased milk production in ewes in late lactation whereas a high dose (5 mg) was inhibitory and decreased milk lactose content from 60 to 45 mg/ml [85]. In explants from midpregnant mice, low concentrations (1 or 5 ng/ml) of 17-β-estradiol, estrone, diethylstilbestrone, but not 17-α-estradiol, stimulated LS and B4GALT1 activity, whereas a high concentration (5 μg/ml) of 17-β-estradiol was inhibitory for LS, but not B4GALT1, activity. The full effect of E on LS activity in these data was only evident 24 h after supplementation with T3 and physiologic levels of PRL or human placental lactogen. Even though the mechanism by which LALBA synthesis is stimulated or inhibited in response to E is unknown, the effect of E on LALBA synthesis in mammary explants was most apparent when the medium was also supplemented with a low, physiologic concentration of PRL [59, 86].

Taken together, different lines of evidence support that E and P can modulate lactose synthesis during the onset of secretory activation and into established lactation. The inhibition of lactose synthesis through the P-induced downregulation of *LALBA* expression is most evident during pregnancy and in the hours immediately following the removal of the P-secreting tissue. Questions linger as to whether P remains inhibitory for lactose synthesis during lactation. The fact that different levels of E biphasically regulate *LALBA* expression is noteworthy and shares similarities with the biphasic response to different levels of GC we outline below. The relationship between E, T3, PRL, and P in the regulation of *LALBA* described thus far underscores the importance of developing *bona fide *ex vivo and in vivo systems for the study of lactose synthesis and milk production.

### Glucocorticoids (GC)

The increasing secretion of cortisol by the adrenal glands during gestation prepares MEC for the onset of copious milk secretion. In fact, GC facilitate an array of cytological changes in MEC including the synthesis of rough endoplasmic reticulum, tight junction closure, increased PRL receptor (PRLR) expression, and regulation of milk protein gene expression [87]. In these ways, mammary explants from midpregnant mice entered a secretory state when exposed to hydrocortisone, corticosterone, or aldosterone at 1 or 5 μg/ml, while deoxycorticosterone was ineffective [88]. The effects of GC are also clearly evident when they are administered to pregnant animals, which invokes secretory activation with or without premature parturition, depending on the species [89–93]. As a case in point, milk lactose concentration and udder distension were increased in multigravid goats within 24 h of a second dose of adrenocorticotropic hormone. Thereafter, the milk lactose content during the rest of the pregnancy did not return to pre-treatment levels, but instead remained elevated at levels seen in mature milk [93]. In a similar way, administering GC to pregnant humans induced secretory activation despite the high circulating level of P, as was evidenced by breast engorgement and increased excretion of urinary lactose [92, 94]. Exogenous GC had less of an effect on the induction of lactose synthesis in humans further along in their pregnancy [91]. Interestingly, ewes that underwent precocious secretory activation in response to exogenous GC subsequently produced less milk with a lower lactose content [90, 91].

There is also a clear impact of GC on milk production during established lactation. Such a relationship is most clear for plasma cortisol, which is negatively associated with milk lactose concentration. However, the association between the concentration of cortisol in milk and its lactose content is less consistent [95–97]. This effect of GC on lactose synthesis, including the effect of exogenous GC, can be revealed in different models and states. For example, lactating humans who received an injection of exogenous GC for musculoskeletal pain had complete or near complete suppression of milk production within one day [98, 99]. In the same way, synthetic GC administered to lactating cows reduced milk lactose concentration from 46 to 43 mg/ml within 24 h of treatment, concomitant with a decrease in milk yield of approximately 10 kg/d [100]. Rat and mouse pups whose dams received daily injections of cortisone had retarded growth within 24 h of treatment [101, 102]. Likewise, hydrocortisone administered to rat dams for the first 15 d of lactation decreased total protein and lactose concentration in milk [103].

Another example of how GC potentially modify the synthesis of lactose can be recorded during times of stress, where both lactose and milk production decrease in association with a dysregulated hypothalamic–pituitary–adrenal axis and increased cortisol secretion [104]. Within 46 h of exposing lactating ewes to a stressful event, lactose content and milk yield decreased, whereas milk fat and protein concentration increased [105]. Similar responses were recorded in dairy cows exposed to transportation stress [106]. Intriguingly, goats did not demonstrate a decrease in lactose synthesis or milk yield following exposure to a stressful event [107–109]. From these data across a range of species it is clear that endogenous GC and high doses of exogenous GC can negatively impact milk production and lactose synthesis.

A primary mechanism underlying the negative effect of GC on milk output likely involves the suppression of LALBA synthesis (Fig. 1), an effect that varies depending on the developmental and lactational stage of the animal as well as the concentration and type of GC. Notably, GC exerted a differential effect on the expression of *LALBA* versus *CSN2* and *B4GALT1*, where low concentrations of GC stimulated *LALBA* expression in explants from midpregnant rats and mice, while high concentrations suppressed *LALBA* synthesis [24, 110, 111]. By contrast, the synthesis of B4GALT1 and CSN2 increased in response to GC in a dose-dependent manner [112, 113]. As a case in point, maximal CSN2 synthesis occurred in response to hydrocortisone concentrations that were 200 times greater than those required for maximal LALBA synthesis [110, 112]. Lactose synthesis within mammary organoids from mice also responded to increasing concentrations of GC in a biphasic manner [24, 114]. For mammary organoids from lactating mice cultured on floating collagen gels, a low concentration of cortisol (0.03 μM) was more stimulatory for LALBA synthesis than a high concentration (3 μM) [115, 116]. Similarly, in mammary explants isolated from lactating cows, deoxycorticosterone at 30 μM inhibited LALBA secretion by 35–45% without affecting glucose uptake [79].

While most of these studies were conducted using tissue or cells from mice, we should point out that other physiological factors likely impact the overall response to GC. For example, the biphasic effect of GC on LALBA synthesis in mammary explants isolated from virgin and midpregnant mice may well not exist for explants isolated from lactating mice [28, 30, 79, 117–119]. Furthermore, the biphasic dose response by LALBA to GC was not observed in a long-term culture system using MEC isolated from either virgin or midpregnant rats [120]. Across these types of experiments there was also variation between individual animals in the amount of LALBA synthesized in response to low, stimulatory concentrations of GC when using mammary explants isolated from late pregnant and lactating rats [28, 30]. These types of variation likely reflect a combination of factors including heterogeneity within the mammary gland, as highlighted above, and interactions with other factors, as outlined below.

Not surprisingly, the effects of GC on LALBA synthesis in vitro are modulated by interactions with other factors including PRL, prostaglandins (PG), T3/T4, or spermidine. One such example is the presence of GC that decreases the dose of PRL required for maximal LALBA synthesis. Specifically, when an inhibitory high concentration of GC was added to cultures along with a lower concentration of PRL (0.5 μg/ml) and INS, the GC-induced suppression of LALBA synthesis was not as pronounced as it was in the presence of a higher concentration of PRL (5 μg/ml) [110]. A similar situation exists for PG, where it reversed the negative effect of high concentrations of GC on LALBA synthesis in cultures of mammary explants from midpregnant mice; the ED_50_ for PGE2, PGF2α, PGA2, and PGB2 to overcome the inhibitory effect of GC were 0.4, 0. 4, 10, and 10 μM, respectively [121]. Notably, PG could not stimulate LALBA synthesis after the GC-induced inhibition was reversed [122]. Unlike PG, T3 not only prevented the negative effect of a high GC concentration on LALBA synthesis, but also stimulated the synthesis of LALBA [73, 122]. Lastly, the production of LALBA in mammary explants from midpregnant mice could be induced without GC when spermidine was added alongside PRL and INS at concentrations as low as 0.4 mM [123]. By contrast, the synthesis of LALBA by explants from midpregnant rabbits required only INS and PRL, but not spermidine or GC, whereas maximal LALBA synthesis in explants from midpregnant rats required the combination of INS, PRL, GC, and spermidine [124].

As outlined above, a role for GC in the regulation of milk synthesis has been dissected extensively in vitro, particularly with regards to its role as a co-regulator of milk protein synthesis. Surprisingly, the extent to which this hormonal modulation occurs in vivo, and the relevance of these findings to lactation and their potential role during environmental exposures such as stress and following the therapeutic use of GC in human and veterinary medicine, remains under-investigated. Of particular relevance to these scenarios is the biphasic regulation of *LALBA* expression by GC, where its negative effect at high levels is likely through its direct effect on lactose and LALBA synthesis.

### Insulin (INS)

Many in vitro studies have cemented the essential role of INS for *LALBA* expression at the level of the mammary epithelium, consistent with its widely-recognized role in stimulating various milk protein genes (Fig. 1) in concert with the effects of PRL and GC. For example, the expression of three genes involved in lactose synthesis, namely *LALBA, UGP2*, and *GLUT1*, increased in response to INS added to cultured mammary tissue from midpregnant mice [125]. Likewise, the expression of *LALBA* mRNA in mammary explants from late-pregnant cows increased tenfold when INS was added to the culture medium [126].

Intriguingly, these robust effects of INS on lactose synthesis in vitro do not translate to a clear indication that plasma INS modulates lactose synthesis in vivo*.* This conclusion aligns with the widespread demonstration that glucose uptake by the mammary glands is INS-independent, consistent with the well-established fact that INS-dependent GLUT4 is absent in mammary tissue [127, 128]. Infusion of INS also did not affect the arteriovenous difference for glucose across the mammary glands of goats, cows, or sheep. In a similar way, milk production and lactose synthesis by cows and sheep was unchanged in response to acute or chronic elevations of plasma INS during a glucose clamp experiment [129, 130]. All these findings are consistent with the fact that a single dose of slow-release INS during the first week postpartum did not affect milk lactose output or milk yield from dairy cows [131].

These differences between the effects of INS on lactose synthesis in vitro and in vivo highlight how considerable gaps still remain in our understanding of both INS action and the regulation of lactose synthesis. Beyond the global role for INS in homeostasis and nutrient partitioning and its dysregulation across a range of conditions, there are still a number of questions that remain regarding its role in support of milk production.

### Β2-adrenergic Receptors and their Downstream Effectors

It is also worth mentioning some of the early studies that examined the ability of signaling downstream of β2-adrenergic receptors to regulate lactose synthesis (Fig. 1). Pregnant rats that received the β1- and β2- antagonist propranolol following the induction of secretory activation had a lower concentration of lactose in their mammary glands, whereas targeting the receptors pharmacologically using either prazosin (an α1 receptor antagonist) or metoprolol (a β1 receptor antagonist) had no effect [132]. By contrast, epinephrine and isoproterenol (β-adrenergic agonists) both inhibited the synthesis of lactose by cultured explants from lactating guinea pigs by 29% and 25%, respectively [27]. These opposing effects of β-adrenergic receptor signaling on lactose synthesis, albeit in two different species and in different physiological states, further highlights the need for a comparative approach to defining the control mechanisms underlying lactose synthesis.

The β2-adrenergic receptors are linked to the adenyl cyclase second messenger pathway (cAMP) and are regulated by PRL and ovarian hormones. The accumulation of LALBA within mammary explants from midpregnant mice decreased by 90% after supplementation with cAMP, whereas the CSN2 content decreased by only 35%. Sodium butyrate, 3’AMP, 5’AMP, adenosine triphosphate, adenosine monophosphate, and guanosine cyclic monophosphate did not affect LALBA synthesis. The inhibitory effect of cAMP on LALBA and CSN2 production was also augmented when a phosphodiesterase inhibitor was present [133]. In a similar way, lactose synthesis by explants from midpregnant mice and lactating guinea pigs was reduced following the supplementation of cultures with cAMP and phosphodiesterase inhibitors [27, 134]. All these findings regarding the effects of β2-adrenergic receptor activation warrant further investigation given the importance of the neuroendocrine system in stress management and the widely-appreciated negative impact of stress on lactation performance.

### Epidermal Growth Factor (EGF)

While EGF plays a crucial role as a paracrine growth factor in the developing mammary glands, there is also strong evidence to support it having a suppressive effect during the onset of lactation. In this way, LALBA activity in cultured explants from midpregnant mice was inhibited by 40% when they were exposed to EGF [135], similar to the suppressive effect of EGF on cultured MEC from lactating mice [116]. Similarly, synthesis of LALBA in ewes, rabbits, and mice was suppressed by EGF in vivo or ex vivo, where ewes in early lactation that received intravenous murine EGF produced less milk with lower lactose content [136]. Likewise, EGF suppressed the induction of *LALBA* by PRL in cultured mammary explants from midpregnant rabbits. Interestingly, this inhibitory effect of EGF was reversed by a low concentration of cortisol that also stimulated LALBA synthesis, whereas corticosterone and aldosterone reversed the suppressive effect of EGF, but were not stimulatory [51]. For reasons that are not entirely clear, the situation in rats appears different, where EGF promoted LALBA synthesis by cultured mammary explants from virgin and midpregnant rats [137]. In keeping with this positive effect, EGF also blocked the inhibition of LALBA synthesis by P in mammary tissue from pregnant rats [137].

### Summary – Hormonal Regulation of LALBA and B4GALT1 Synthesis

Taken together, it is perhaps not surprising that a milieu of hormones and their interactions can dramatically modulate lactose synthesis, which is achieved in a large part at the level of LALBA transcription. Many of these findings are based on some very detailed and thorough in vitro studies, particularly using mammary explants and relatively defined conditions. In our view, the physiological implications of these data are yet to be fully captured, whether that be for identifying ways to improve breastfeeding success, optimize milk production for dairy livestock, or support neonatal growth.

## The Genetic Regulation of Lactose Synthesis

In the previous section we detailed the impact of endocrine signals on lactose synthesis, particularly through their ability to positively or negatively affect *LALBA* expression. The nature of this regulation is, of course, particularly relevant during reproductive progression, as well as during adverse states such as stress. However, the synthesis of lactose is also determined at the genetic level, which applies across a range of taxonomic groups. Here we summarize a range of genetic mechanisms that directly regulate, or are associated with, altered lactose synthesis across numerous species and systems, with a primary focus on the genetic regulation of LALBA and B4GALT1.

### Polymorphisms in Genes Outside the Lactose Synthesis Pathway

The ability to screen for genetic polymorphisms in livestock including cattle, sheep, and horses has led to the identification of various genomic variants that are associated with measurable alterations in lactose output. In many cases, not surprisingly, these variants can be implicated in pathways underlying the synthesis of other major milk components including $\beta$-lactoglobulin [138–142], milk fat (1-acylglycerol-3-phosphate O-acyltransferase 6 and diacylglycerol O-acyltransferase 1) [143–145], lactotransferrin [146] and the caseins [146]. In other cases, polymorphisms are more directly implicated in the hormonal regulation of the synthesis of lactose or other milk components, as is the case for the leptin receptor [143, 147, 148], growth hormone [146], growth hormone receptor, PRL, and suppressor of cytokine signaling 3 [138–142], or glucocorticoid receptor DNA-binding factor-1 [146] genes. While associative, these types of analyses can inform genetic selection strategies in livestock, where similar data accompanied by lactation performance measures will undoubtedly reveal a better understanding of the genetic regulation of lactose synthesis in humans.

### Genetic Variation in B4GALT1 and its Impact on Lactose Synthesis

The B4GALT genes are expressed by most cell types to support intracellular glycosylation. By contrast, *B4GALT1* expression in MEC is tightly regulated during gestation and lactation to coordinate with, and support, lactose synthesis. Until mid-pregnancy, MEC transcribe a 4.1 kb *B4GALT1* mRNA with a 175 nucleotide 5’ untranslated region (5’ UTR), concurrent with binding of Sp1 immediately upstream of a transcription start site (TSS). Subsequently, during late-pregnancy and throughout lactation, specificity factor 1, nuclear factor 1/CCAAT box-binding transcription factor, and Apetala 2 bind a different region, either ~200 bp upstream or downstream from the same TSS, yielding a truncated 3.9 kb mRNA transcript. This 3.9 kb mRNA transcript has a shorter 5’UTR that lacks an extensive secondary structure and has increased translational efficiency [1, 149, 150].

Several SNP exist within the bovine *B4GALT1* gene. Among nine SNP, three were associated with lower lactose content in milk whereas three others were associated with higher lactose content. Consistent with the aforementioned modulation of B4GALT1 mRNAs, one of these SNP was in the TSS and directed the switch between the long and short form of the *B4GALT1* 5’UTR in association with the milk having a lower lactose content. Two SNP were present in the B4GALT1 catalytic domain and were associated with a higher lactose content in milk. While SNP also exist within the region of B4GALT1 that interacts with LALBA, none were significantly associated with milk composition or volume [151].

### Regulation of LALBA Gene Transcription

Given the critical role of lactose across a broad range of mammals, it is not surprising that the genetic structure of *LALBA* is widely-conserved, including its exon–intron boundaries [152–157]. The first three exons of *LALBA* are homologous to the lysozyme gene, while the fourth is unique [152]. In a similar way, a comparative analysis of the regulatory factor binding sites located in the *LALBA* promoter in the bovine, caprine, human, murine, rat, and swine genomes revealed three conserved motifs (LA1, LA2, LA3) located in the proximal end of the promoter sequences that were distinct from motifs found in the promoters of other milk protein genes [158].

The pronounced change in LALBA mRNA abundance during pregnancy and into lactation highlights how tightly its expression is coordinated at the transcriptional level. The murine *LALBA* proximal promoter (~ 2.5 kb upstream of the TSS) has an open chromatin structure across all reproductive states [159, 160]. Surprisingly, the binding of only a few transcription factors to the *LALBA* promoter has been assessed. The LALBA gene in rats and humans, as well as their five casein genes, all share an nuclear factor 1 binding site in their proximal promoter [161]. The promoters for mouse, rat, human, and bovine *LALBA*, as well as the Ca-sensitive caseins and whey acidic protein, also have a conserved STAT5 binding site. Within the human *LALBA* promoter, these STAT5 binding sites are all proximal to steroid hormone binding sites [162, 163]. Additional repeated hexanucleotide sequences have also been identified in the human and rat *LALBA* promoter, although they do not resemble the consensus GR response element [153]. Consistent with this genomic landscape, both GR and pSTAT5 were bound to the murine *LALBA* promoter on days 1 and 10 of lactation [48]. Despite the fact that P clearly regulates lactose synthesis with the onset of lactation, it has not been established whether the *LALBA* promoter has a P receptor binding site in its 5’UTR [155]. Interestingly, the TATA, CCATT, GC response element boxes, and mammary gland-specific transcription factor sequences were not identified in a 500 bp region upstream of the tammar wallaby *LALBA* coding sequence [164], perhaps reflecting the differential control of lactational output across developmental stage in this species.

In addition to regulation at the promoter, *LALBA* transcription is also influenced by its distal enhancer, which lies 1500 bp upstream of the bovine *LALBA* TSS [165]. This region is 75% homologous to the *CSN2* distal enhancer. While the *CSN2* distal enhancer has consensus binding sites for pSTAT5 and C/EBP, the transcription factors that bind the putative *LALBA* enhancer are yet to be defined [166, 167], although GR and pSTAT5 were bound to the putative murine *LALBA* super-enhancer on L(1) and L(10) [48].

Superimposed on these transcriptional controls is an epigenetic landscape for the *LALBA* gene that is distinct from that for *CSN2* or whey acidic protein. In mice, the *LALBA* proximal promoter has an open chromatin structure across all reproductive states, which supports the notion that fine-tuning of *LALBA* transcription primarily occurs through the binding and tethering of transcription factor complexes to its proximal promoter [160]. The tailoring of an epigenetic environment in support of lactose synthesis is also illustrated by the fact that the different genes that contribute to lactose synthesis all consistently maintain the chromatin modifications they acquired during pregnancy and lactation. By contrast, the epigenetic modifications surrounding the *CSN2* gene reverted to their pre-gestational state after involution [168].

### Genomic Variation and the Regulation of LALBA Function

The considerable genetic variation that exists within the LALBA gene across species also offers potential insights to its core functional elements. At the nucleotide level, there is a multitude of SNP within both the 5'UTR and coding regions of the *LALBA* gene, although few have been analyzed for their association with milk yield or composition [165, 169–177]. At one extreme, a single SNP 15 bp away from the *LALBA* TSS in Holstein cows was associated with higher lactose content and milk yield, but lower fat and protein content, and was proposed to account for a 30-fold greater expression of LALBA in explants from Holstein versus Angus cows [171, 178, 179]. Intriguingly, the same SNP in Swedish Red and White cows did not affect milk lactose concentration [178]. In a similar way, an I/V substitution at amino acid 46, the site of LALBA glycosylation, did not affect LALBA or lactose concentration in human milk [170], and none of four SNP in the 5’UTR of the equine *LALBA* mRNA were associated with altered LALBA mRNA or protein expression [177]. Among Chinese Holstein dairy cows, a T1847C SNP in a noncoding region was associated with lower lactose content and yield, but not fat or protein content [180]. Nine SNP were identified in the 5’UTR and 3’UTR of the Sarda goat *LALBA* mRNA transcripts, of which two SNP (-368 and -163) located at apetala 2α and specific factor 1 transcription factor binding sites, respectively, were associated with lower milk lactose content [181].

What is perhaps even more enlightening is the genetic and associated phenotypic variation that exists within the LALBA gene across various marine mammals. The LALBA promoter in the Cape fur seal has a series of *cis-*acting mutations that results in the synthesis of a viscous, lactose-free milk with a high concentration of protein and fat [182]. In the California sea lion, the Antarctic fur seal, and the Cape fur seal, the *LALBA* TATA box has a T-G transversion (AAGAAA) in the third position that prevents binding of the TATA binding protein, thereby preventing transcription initiation. However, the introduction of a STAT5 binding site and correction of the transversion in the TATA box in the LALBA promoter for the Cape fur seal did not activate gene transcription, suggesting that other mutations, like the disruption in the fourth exon found in the otariid LALBA gene, also contribute to the inability of the Cape Fur seal to synthesize LALBA and lactose [182, 183]. Interestingly, the Atlantic walrus has a 7 bp deletion that leads to a frame shift in exon 4 of *LALBA*, which translates to a longer 176 amino acid protein that is incapable of participating in lactose synthesis [183].

Taken together, these multiple layers of genomic and transcriptional regulation highlight how the genetic basis of lactose output has evolved as a tightly-coordinated program, while also being semi-independent from the expression of other milk proteins. There is also a great deal that remains to be learned about how these transcriptional controls are regulated and coordinated, not only across the lactational cycle, but also within individual cells and regions within the gland. Regardless, the combination of these insights points to a vast opportunity to harness and optimize these regulatory mechanisms, whether it be to manipulate milk composition or to improve the milk production potential in humans and livestock.

### Post-translational Control of LALBA

The LALBA mRNA and protein undergo significant post-transcriptional and post-translational regulation and processing [184]. The primary site of LALBA glycosylation surrounds the N-glycosylation consensus sequence at Asn-45 [185, 186], where glycosylation has been proposed to suppress the secretion of LALBA to allow for quality control at the level of the endoplasmic reticulum [187]. How the extent or nature of LALBA glycosylation impacts lactose synthesis and milk output is unclear, as we alluded to previously [1]. Introducing an Asn45Asp substitution into the water buffalo LALBA rendered it incapable of being glycosylated, although the associated milk composition was unchanged [186]. Goat LALBA contains two glycosylated residues at amino acids 45 and 74, yielding either an unglycosylated, singly- or doubly-glycosylated molecule [187]. Secretion of goat LALBA in a yeast culture system was suppressed when the number of N-linked glycosylation sites was increased to three, whereas its secretion was highest when amino acid 45 was mutated and N-linked glycosylation was lost [187]. Certainly, there are physiological contexts where glycosylation of LALBA also varies. For example, adding EGF to explants from midpregnant rats cultured with INS, PRL, and GC decreased the synthesis of glycosylated LALBA by approximately 30%, such that the ratio of the two forms was 1:1 [188]. Conversely, supplementing cultures with T3 increased the abundance of glycosylated LALBA, whereas only non-glycosylated LALBA was produced by explants cultured in its absence [189].

### Lessons from Transgenic Animals Carrying an Exogenous LALBA Sequence

Transgenesis has served as a particularly innovative and insightful means to study and manipulate different aspects of the lactose synthesis pathway in animal models. We have elected to review those studies here, rather than in the respective sections above, because it is important to recognize that the context of situations like overexpression, heterologous systems, and altered physiological function can lead to different outcomes that may cloud any interpretations.

For some time a standing assumption was that the *LALBA* proximal promoter was sufficient to direct maximum gene expression, whereas optimal transcription of *CSN*2 required its distal enhancer elements [165, 190]. In early experiments, only short (< 1 kb) *LALBA* promoter fragments were used to direct transgene expression in mice, based on the knowledge that many important, albeit undefined, *cis*-acting elements are located between positions -477 and -220 [191]. Transgenic mice with a longer 5’ *LALBA* promoter fragment expressed bovine LALBA at approximately 1000 times higher concentrations than those harboring a shorter 5’ fragment. While the resultant milk lactose content was not measured, transgenic mice that expressed higher quantities of bovine LALBA produced viscous milk [192]. When a 2 kb *LALBA* promoter was used to direct the expression of bovine CSN2 in transgenic mice, the MEC underwent premature involution in association with more production of CSN2 and a viscous milk, similar to that described in *LALBA* knock out mice [193]. These findings contrasted with the phenotype of transgenic mice expressing caprine ß-casein under the control of the caprine κ-casein promoter that maintained their milk production and composition. The authors proposed that the bovine LALBA 5’UTR sequestered transcription factors from the endogenous LALBA promoter, suppressing the production of LALBA and lactose [190, 193, 194].

Interestingly, a range of transgenic animal models has supported the general conclusion that overexpression of exogenous LALBA differently affects milk lactose content across species. Transgenic mice overexpressing human *LALBA* from a construct containing a 0.77 kb 5’ fragment expressed the exogenous gene and protein at levels 14-fold greater than those for endogenous LALBA, without any effect on milk lactose content [195]. Transgenic sows bearing a bovine *LALBA* construct that included 2 kb of upstream sequence produced 20–50% more milk that had a higher milk lactose content and lower total solids, protein, and fat concentration than control animals [50]. This positive effect of bovine *LALBA* on milk composition was still apparent in the second lactation, where sows produced twice the amount of bovine LALBA in colostrum and milk versus during their first lactation [196, 197]. The concentration of bovine LALBA in transgenic mice varied tenfold between mice from the same transgenic line, suggesting that variation in the expression of exogenous LALBA was not just due to random integration of the transgene into the genome [192]. Likewise, the amount of human LALBA secreted into milk from transgenic cows varied from 0.17 to 1.56 mg/ml [198]. Transgenic cows only produced unglycosylated human LALBA, whereas transgenic mice produced bovine LALBA that was glycosylated at levels similar to those found in bovine milk [198, 199]. Transgenic cows expressing human *LALBA* also expressed 43 unique proteins in the milk fat globule membrane without any apparent effect on the biology of milk synthesis [200].

These various animal experiments highlight the potential importance of regulatory elements within the 5’ UTR of the *LALBA* gene. Combined with the aforementioned transcriptional regulatory mechanisms, it becomes clear that there is a host of conserved as well as species-specific regulatory elements that control and optimize *LALBA* transcription. These findings also set the stage for future, more precise genetic modification strategies, such as those that can be edited using CRISPR/Cas9.

## Conclusion

In this review we focused on defining the range of control points that regulate lactose synthesis, particularly at the endocrine and genetic levels. As we outlined above, combinations of intracellular and intramammary regulatory factors (Fig. 1) are among the primary control points for lactose synthesis, more so than extramammary conditions like plasma glucose and blood flow. Nevertheless, dysregulation in the delivery of plasma glucose is inextricably tied to lactational output and is associated with stress and metabolic syndromes, such as obesity and diabetes mellitus. Plasma glucose availability and its uptake by the mammary gland for lactose synthesis is also modulated by the negative effect of fasting, caloric deprivation, and dietary carbohydrate restriction. Moving forward, one consideration is that lactating rodents may not be the best translational model for the study of food deprivation on lactose synthesis given their response is much more pronounced than that for lactating humans, and that they do not recapitulate the lower plasma glucose levels seen in lactating ruminants.

In considering the crucial role for, and regulation of, lactose, there is no doubt that its synthesis and function(s) are a centerpiece for a range of emerging scientific concepts and global issues. Lactose plays a vital role in the movement of water which is a major component in dairy products worldwide. All these processes, as well as the survival of threatened species across a warming planet, depend on the movement of ever-scarcer water that is facilitated by the actions of lactose. At the same time, LALBA and lactose are critical for infant nutrition, as sources of protein and carbohydrate, respectively. Lactose also serves as the building block for a range of oligosaccharides that we now recognize have critical roles in regulating infant growth and development via the gastrointestinal microbiome.

With advances in genetic engineering and selection, there may also be ongoing opportunities to manipulate milk composition by targeting the lactose synthesis pathway. As a starting point, genetic mutations in the *LALBA* promoter that directly lead to a reduction in lactose synthesis need to be defined. Furthermore, the transcriptional regulators within the promoter and enhancer regions of the *LALBA* gene require better resolution as a way to screen and risk-stratify patients by their need for additional lactation support services or tailored therapeutic regimens. Many of these questions can now be pursued using mainstream sequencing technologies and non-invasive methods of studying the transcriptome from cells and the milk fat globule in milk. Special attention should also be placed on the species-specific effects of PRL, EGF, and thyroid hormone and the biphasic regulation of LALBA by E and GC, given that both steroids are involved in endogenous physiological responses and are common pharmacologic agents used in human and veterinary medicine. All these questions become additionally challenging to study given that there is an ongoing absence of in vitro models that mimic lactose synthesis and secretion, which hinders progress in the field. This issue of optimized models for milk synthesis in vitro becomes an important area for reconciliation that would have a significant translational impact across a range of applications.

## Supplementary Information

Below is the link to the electronic supplementary material.

Supplementary file1 (PDF 59 KB)

## Data Availability

Data will be made available by the corresponding author upon request.

## Abbreviations

LALBA: α-Lactalbumin
B4GALT1: β-1,4-Galactosyltransferase-1 gene
CSN2: β-Casein
D: Day
EGF: Epidermal growth factor
E: Estrogen
GC: Glucocorticoid
GR: Glucocorticoid receptor
GLUT1: Glucose transporter 1
h: Hour
INS: Insulin
LII: Lactogenesis II
LS: Lactose synthase
MEC: Mammary epithelial cell
min: Minute
PRL: Prolactin
PRLR: PRL receptor
P: Progesterone
PG: Prostaglandin
STAT5: Signal tranducer and activator of transcription 5
SNP: Single nucleotide polymorphism
TRH: Thyroid hormone releasing hormone
T4: Thyroxine
T3: Trioiodothyronine
TSS: Transcription start site
UTR: Untranslated region
